# Supplementary material for: Pen drawing display
Source: Nat Commun. 2019 Sep 24;10:4334. doi: 10.1038/s41467-019-12395-z (PMC6760158; doi:10.1038/s41467-019-12395-z)
Supplement: Supplementary file 1 — Supplementary Information [file 41467_2019_12395_MOESM1_ESM.pdf]

Supplementary Information for

Pen Drawing Display

Sang-Mi Jeong et al.

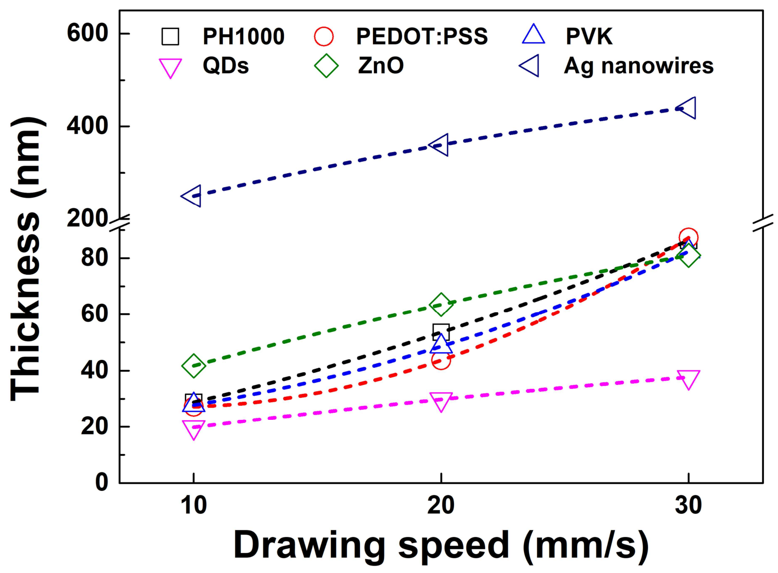

Supplementary Figure 1. Classical Landau–Levich model. When the pen drawing speed increases, the film thickness of each layer constituting the pen drawing display structure also increases.

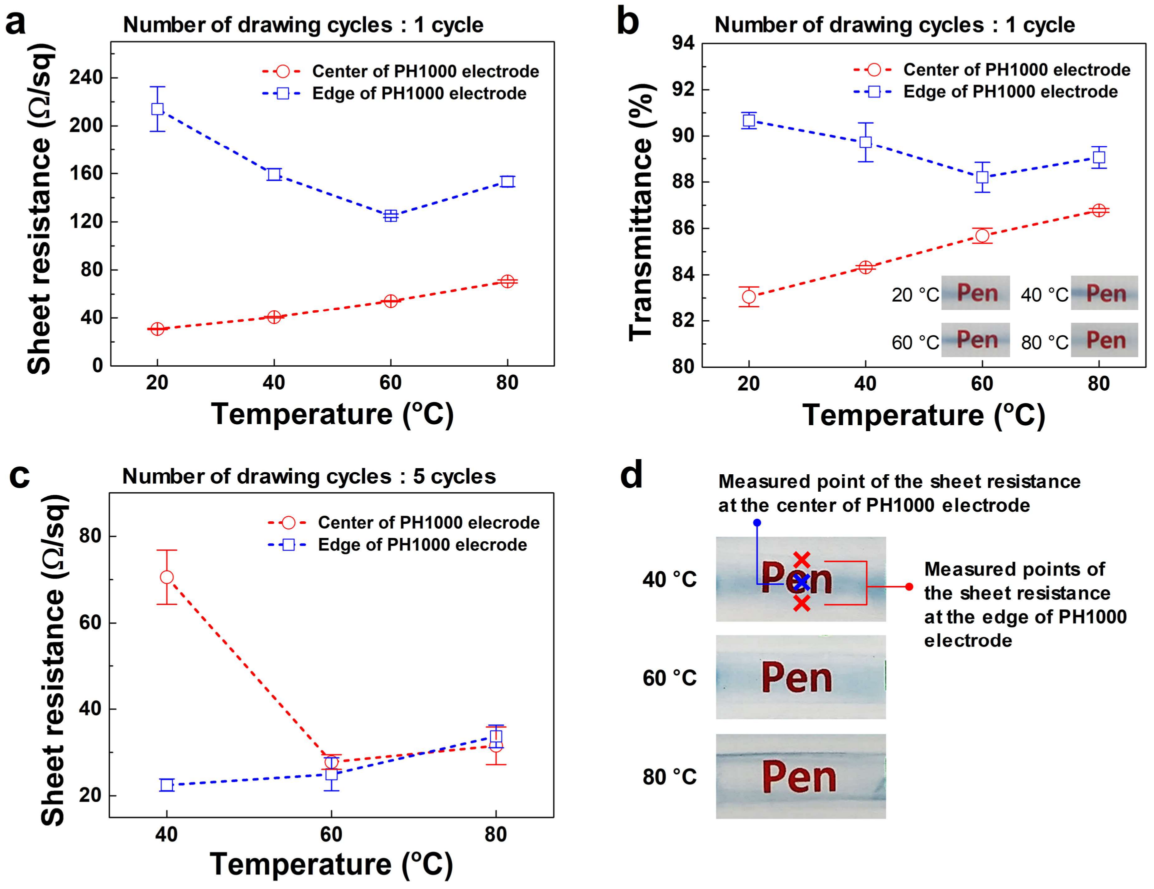

Supplementary Figure 2. Optical and electrical properties of the PH1000 layer. The substrate temperature at a drawing speed 20 mm/s. Sheet resistance (**a**) and optical transmittance (**b**) at the center and edge of the PH1000 film at substrate temperatures of 20, 40, 60, and 80 °C in the case of 1 drawing cycle. (**c**) Sheet resistance at the center and edge of the PH1000 film at substrate temperatures of 40, 60, and 80 °C in the case of 5 drawing cycles. (**d**) Measured points of sheet resistance at the center and edge of the PH1000 layer at substrate temperatures of 40, 60, and 80 °C.

The substrate temperature was controlled to form a uniform film of the PH1000 layer, which is the anode, using the pen drawing method. Supplementary Figures 2a and 2b show the sheet resistance

and transparency results when the substrate temperature was 20, 40, 60, and 80 °C under a fixed drawing speed of 20 mm/s and 1 drawing cycle. As the temperature increased, the difference in the sheet resistance and transparency between the center and the edge of the PH1000 film gradually decreased. However, a uniform film was not formed even when the substrate temperature was increased to 80 °C, as the difference in the sheet resistance between the center and the edge of the layer was ~80 Ω/sq and the difference in the transparency was ~3%. The insets in Supplementary Figure 2b show images of the PH1000 layer on a glass substrate formed at different temperatures placed on paper on which the text "Pen" was printed.

Supplementary Figures 2c and 2d show the result of forming PH1000 layers at different substrate temperatures (40, 60, and 80 °C) with 5 drawing cycles. At a substrate temperature of 60 °C, the difference in the sheet resistance between the center and the edge of the PH1000 layer was uniform at ~3 Ω/sq. Furthermore, Supplementary Figure 2d also shows a uniform PH1000 layer. However, when the substrate temperature was increased to 80 °C, rapid drying occurred at the edge of the wet film and a coffee-ring effect was generated, whereby the solutes moved to and accumulated at the edge by capillary flow.

To verify the uniformity of the PH1000 layer, the sheet resistance and transparency were measured separately at the center and edge of the layer. The width of the layer formed with a felt-tip pen was ~4 mm. The edge was determined to be 1 mm from the top and bottom. The center and edge were measured at least five times, and the average and standard deviation values were calculated (Supplementary Figure 2d).

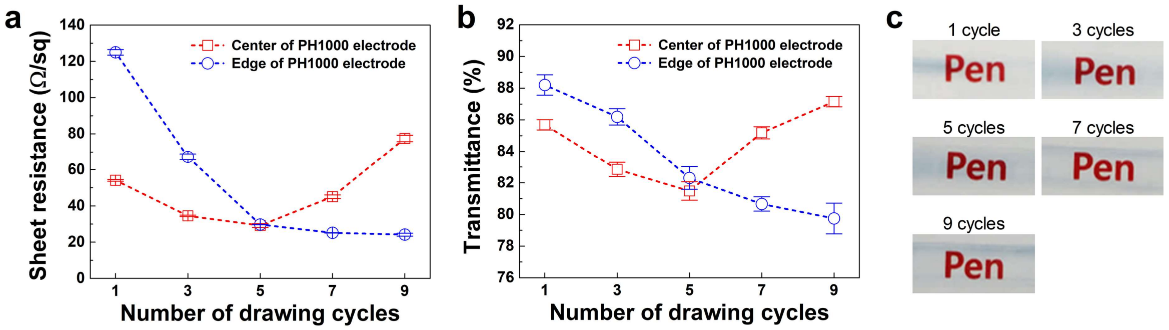

Supplementary Figure 3. Optical and electrical properties of the PH1000 layer. The number of drawing cycles at a drawing speed of 20 mm/s and a substrate temperature of 60 °C. Sheet resistance (**a**) and optical transmittance (**b**) characteristics of the PH1000 layer measured at the center and edge. (**c**) Optical images of the PH1000 layer according to the number of drawing cycles.

Supplementary Figure 3 shows the distributions of the sheet resistance and transparency at the center and edge of the PH1000 film formed while changing the number of drawing cycles (1, 3, 5, 7, and 9) under a drawing speed of 20 mm/s and a substrate temperature of 60 °C. When the number of drawing cycles was small, the hydrophobic PEDOT molecules of PH1000 were agglomerated at the center owing to hydrophilic–hydrophobic repulsion with the glass substrate while the PH1000 wet film on the hydrophilic glass substrate was drying. This behavior resulted in a large difference in the sheet resistance and transparency between the center and the edge. However, as the number of drawing cycles was increased, uniform sheet resistance and transparency were observed at both the center and the edge, especially after 5 cycles, because the PH1000 wet film formed by pen drawing was spread evenly by the shear force until the film was dried and the viscosity increased. However, when the number of drawing cycles was further increased to 7 and

9, additional PH1000 solution was applied to the dried film at the edge, and the difference in the sheet resistance and transparency between the center and the edge increased.

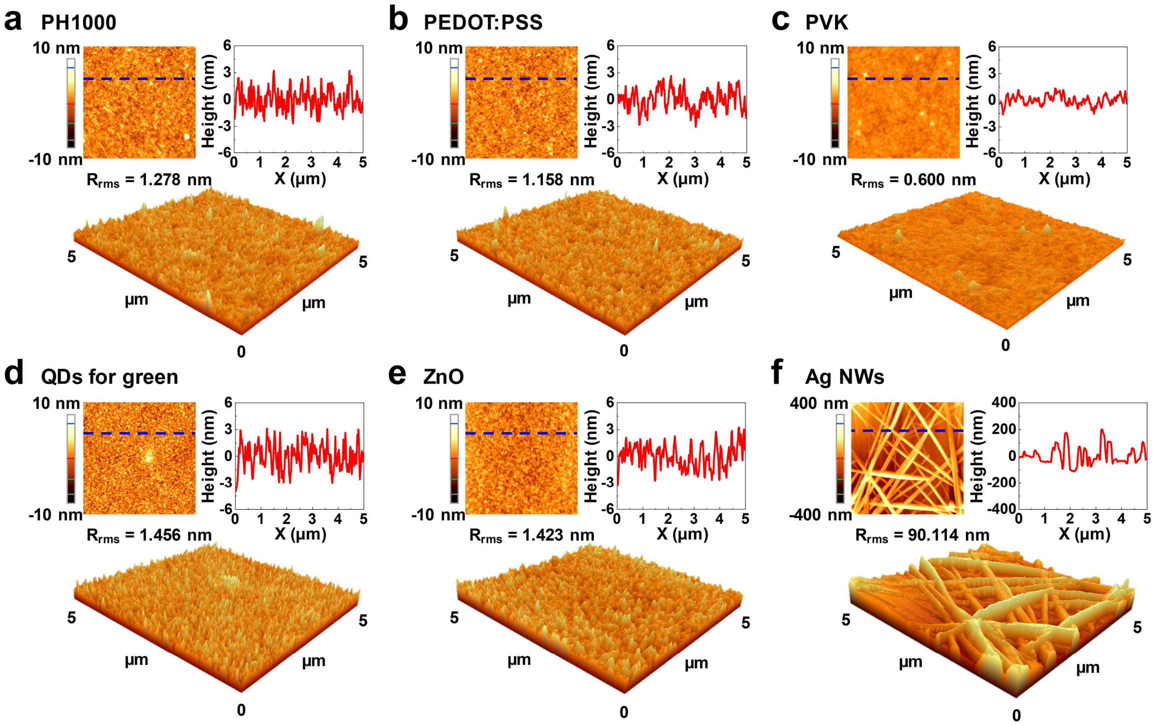

Supplementary Figure 4. Surface morphology of each layer formed by spin coating. The atomic force microscopy (AFM) topographic images, height section analyses, and three-dimensional images of PH1000 (**a**), PEDOT:PSS (**b**), poly(*N*-vinylcarbazole) (PVK) (**c**), quantum dots (QDs) for green (G) (**d**), zinc oxide (ZnO) (**e**), and silver nanowires (Ag NWs) (**f**).

Supplementary Figure 4 shows the surface morphology results for the thin films of PH1000 (3000 rpm, 60 s), PEDOT:PSS (3000 rpm, 60 s), PVK (3000 rpm, 60 s), QDs for green (G, 3000 rpm, 20 s), zinc oxide (ZnO, 3000 rpm, 60 s), and silver nanowires (Ag NWs, 3000 rpm, 60 s) formed on the glass substrate by the spin-coating method. The resultant root mean square (RMS) roughness (R_{rms}) values of PH1000, PEDOT:PSS, PVK, QDs (G), ZnO, and Ag NWs layers were 1.278, 1.158, 0.600, 1.456, 1.423, and 90.114 nm, respectively.

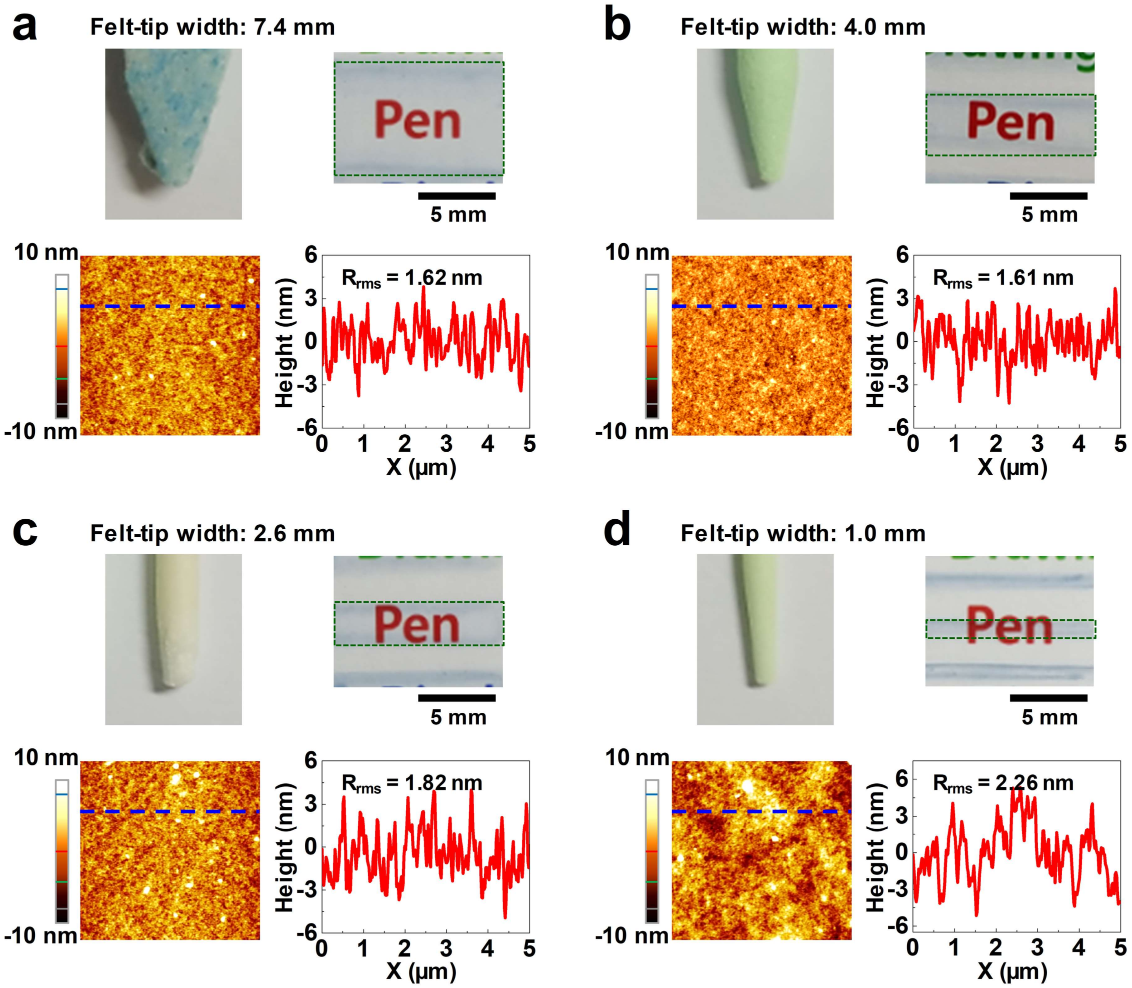

Supplementary Figure 5. Line images and surface roughness properties of the PH1000 layer. The PH1000 layer formed by felt-tip pens with different pen tip widths: 7.4 (**a**), 4.0 (**b**), 2.6 (**c**), and 1.0 (**d**) mm.

In addition to the felt-tip pen used for bold text (width, 4.0 mm; DONG-A, Twinliner SOFT) in this study, felt-tip pens with widths of 7.4, 2.6, and 1.0 mm were used. The PH1000 films were formed on a plastic substrate using the following drawing conditions: 5 cycles of pen drawing, a drawing speed of 20 mm/s, and a substrate temperature of 60 °C. Here, the line widths of the layers

formed by pen drawing were 7.5, 4.0, 2.7, and 1.2 mm, which were almost identical to the widths of the felt-tip pens used (7.4, 4.0, 2.6, and 1.0 mm, respectively). Therefore, a user can form a desired line width on a display by drawing the required area using either multiple lines with a thin felt-tip or a single line with a thick felt-tip. Furthermore, the layer roughness determined through atomic force microscopy (AFM) measurements (R_{rms} values of 1.62, 1.61, 1.82, and 2.26 nm for felt-tip pen widths of 7.4, 4.0, 2.6, and 1.0 mm, respectively) show similar results (Supplementary Figure 5). In this study, the layer-formation images and transmittance and sheet resistance results correspond to the averages and standard deviations for 10 repeated measurements. Accordingly, it was ascertained that reliable layer formation and QD-LED fabrication could be facilitated through pen drawing.

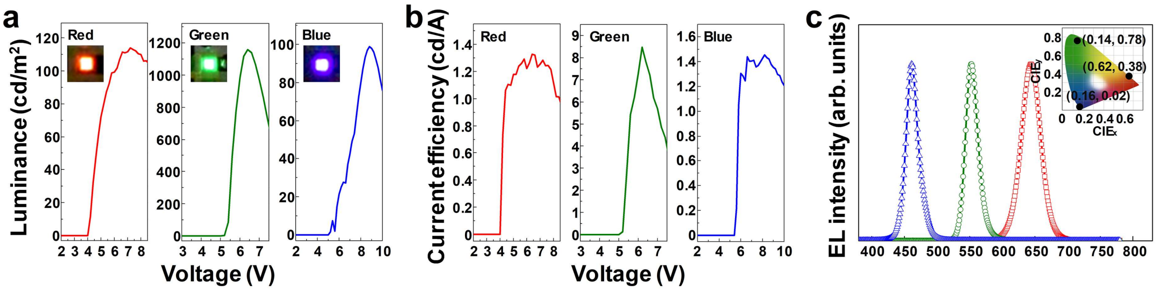

Supplementary Figure 6. Luminance–current–voltage (LIV) characteristics. Luminance (a), efficiency (b), and EL spectra and the Commission Internationale de l'Elcairage (CIE) color coordinates (**c**) corresponding to the red (R), G, and blue (B) emitting quantum dot light-emitting diodes (QD-LEDs) with the structure of indium-tin-oxide (ITO)/PEDOT:PSS/PVK/QDs (CdSeS@ZnS for R, CdZnSeS@ZnS for G, and CdZnS@ZnS for B)/ZnO NPs/aluminum (Al).

Supplementary Figures 6a–c show the luminescence–current–voltage (LIV) characteristics of the QD-LEDs that showed red (R), G, and blue (B) emission. For these QD-LEDs, the hole injection layer, hole transfer layer, emission layer, and electron transfer layer were produced by pen drawing, whereas the anode and cathode layers were deposited by sputtering with indium-tin-oxide (ITO, ~100 nm) and aluminum (Al, ~150 nm), respectively. The luminescence characteristics of the QD-LEDs showed turn-on voltages of ~4 (R), ~5 (G), ~5 (B) V and maximum luminance values of 114 (R), 1158 (G), and 99 (B) cd/m^2 at 7.2, 6.4, and 8.8 V, respectively. The current efficiencies were 1.3 (R), 8.5 (G), and 1.5 (B) cd/A at 6.4, 6.2, and 8.2 V, respectively. The electroluminescence (EL) spectrum of the QD-LEDs had maximum peaks of ~640 (R), ~550 (G), and ~460 (B) nm, respectively. As shown in the inset of Supplementary Figure 6c, the QD-LEDs

showed the Commission Internationale de l'Elcairage (CIE) color coordinates of (0.62, 0.38) (R), (0.14, 0.78) (G), and (0.16, 0.02) (B).

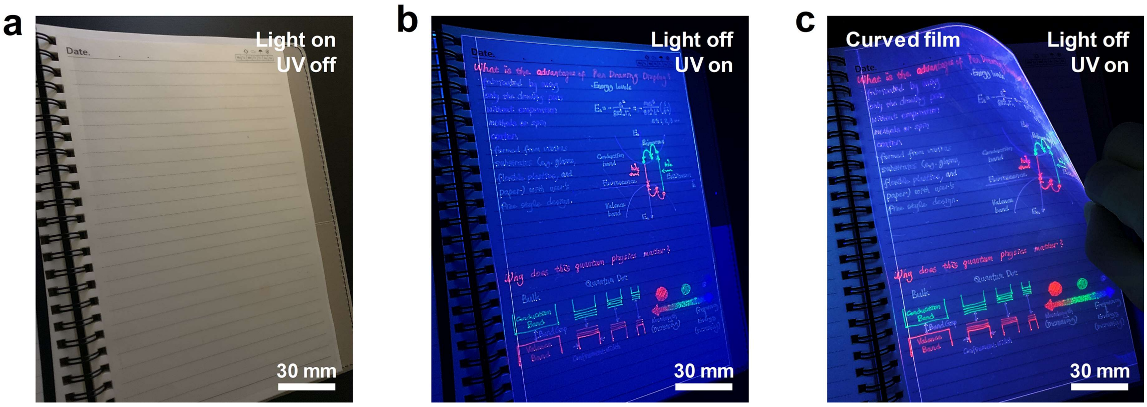

Supplementary Figure 7. Images of a various letters and figures handwritten on a flexible substrate. Images of the handwritten substrate captured under visible light (a) and ultraviolet (UV) light (b). (c) Image of the curved film captured under UV light.

Notes were created on a transparent, flexible overhead transparency film (OHP film; 210 × 297 mm^2; thickness, 0.12 mm; 3M PP 2910) using the pen drawing method with RGB QD solutions and QD solutions (white, yellow, and sky blue) produced by mixing the RGB QD solutions. The notes were handwritten like actual lecture notes using two types of felt-tip pens with felt-tip sizes of 4 and 0.4 mm. Supplementary Figure 7a shows an image of the transparent plastic substrate with handwriting produced by pen drawing using the QD solutions exposed to normal visible light. When the substrate was illuminated with ultraviolet light in a slightly dark state, the letters and figures produced by pen drawing with the QD solutions appeared vividly (Supplementary Figure 7b). Furthermore, as shown in Supplementary Figure 7c, the pen-drawn letters and images appeared vividly even when the plastic substrate was bent.
